# Paediatric vitreous haemorrhage secondary to clinically occult, anomalous hyaloid and peripapillary arteries demonstrated by OCT-A

**DOI:** 10.1038/s41433-021-01852-z

**Published:** 2022-02-17

**Authors:** Imran H. Yusuf, Amanda Ie, Ravi Purohit, Sarah Maling, Chetan K. Patel

**Affiliations:** 1 Nuffield Laboratory of Ophthalmology, Department of Clinical Neurosciences, University of Oxford, John Radcliffe Hospital, Oxford, UK; 2 Oxford Eye Hospital, John Radcliffe Hospital, Oxford University Hospitals NHS Foundation Trust, Oxford, UK; 3 Stoke Mandeville Hospital, Buckinghamshire Healthcare NHS Trust, Mandeville Road, Aylesbury, HP21 8AL UK

**Keywords:** Retinal diseases, Optic nerve diseases

Spontaneous, non-traumatic paediatric vitreous haemorrhage may occur secondary to a persistent hyaloid artery, which is easily diagnosed if visible on fundoscopy. We present two cases of unexplained paediatric vitreous haemorrhage, referred to our tertiary clinic, in which optical coherence tomography angiography (OCT-A) was used to demonstrate flow through otherwise clinically occult, hyaloid and peripapillary arterial remnants. OCT-A may prevent the need for extensive investigations in some cases paediatric vitreous haemorrhage, previously classified as idiopathic.

## Case 1

**Fig. 1:**
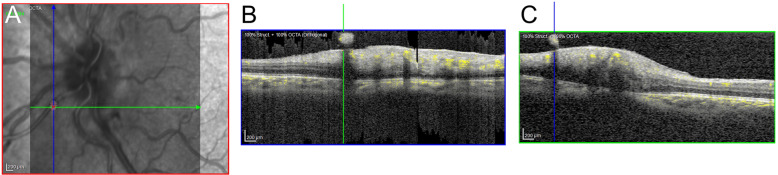
OCT-A imaging in Case 1. **A** En-face infra-red image of the optic nerve head captured on Heidelberg Flex, with an overlay (red) showing flow anterior to the internal limiting membrane. **B**, **C** Vertical and horizontal OCT-A cross-sections intersecting the area of flow in the vessel protruding into the vitreous.

A 12-year-old female was referred with sudden loss of vision in her left eye to “perception of light”. Visual acuity in the right eye was 6/6 with correction. A fundus obscuring vitreous haemorrhage was present. Dynamic B-scan ultrasound confirmed a flat retina with complete posterior vitreous detachment. Examination of the fellow eye was within normal limits. There was no significant ocular or family history. The patient underwent a diagnostic and therapeutic vitrectomy. An underlying cause was not identified. Intraoperative fundus fluorescein angiography of the fellow eye was normal. At post-operative review, her visual acuity was 6/7.5 OS with correction. OCT-A demonstrated an artery with flow signal in the left eye extending into the vitreous cavity located 1.1 mm inferonasal to the centre of the optic (Fig. 1). The vitreous haemorrhage did not recur.

## Case 2

**Fig. 2:**
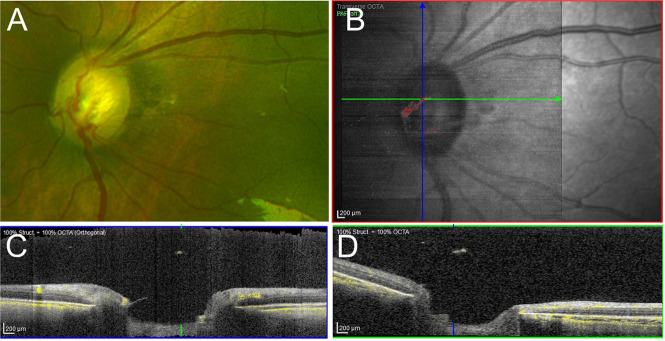
OCT-A imaging in Case 2. **A** En-face image of the optic nerve head using Optos imaging. No obvious anomaly was noted. **B** En-face infra-red image captured using Heidelberg Flex, with an OCT-A overlay showing the areas where flow was identified, (illustrated in red). **C**, **D** Vertical and horizontal OCT-A cross sections (with reference to Fig. 2B) showing flow (yellow) in the hyaloid remnant.

A 6-month-old male infant of African heritage presented at 46 weeks corrected gestational age on a background of prematurity (23 + 1 weeks), with a birth weight of 550 g. He previously underwent bilateral laser photocoagulation for retinopathy of prematurity at 31 weeks postmenstrual age. He then developed a pre-retinal haemorrhage in the right eye. Examination under anaesthesia with conventional fluorescein angiography demonstrated areas of temporal nonperfusion in both eyes; bilateral laser photocoagulation was performed. Avulsed retinal vessels were present in both eyes with regressed stage 3 disease, without plus disease. Complete resolution of the vitreous haemorrhage was noted one week post-operatively. OCT-A was subsequently performed, which revealed the presence of a bilateral clinically occult persistent hyaloid artery (Fig. 2).

## Discussion

Spontaneous and traumatic paediatric vitreous haemorrhage secondary to persistent hyaloid artery remnants have been well described [1, 2]. Here we demonstrate the presence of clinically occult, anomalous retinal arteries in two children presenting with vitreous or pre-retinal haemorrhages, with flow signals demonstrated using OCT-A.

A non-systematic review identified that approximately 1.7–6.5% of cases of spontaneous, unilateral paediatric vitreous haemorrhage are idiopathic [3]. It is possible that clinically occult, persistent anomalous artery remnants may explain some of these cases. Whilst OCT-A may demonstrate flow in clinically obvious hyaloid arteries [4], we recommend OCT-A of the optic disc for paediatric patients presenting with unexplained vitreous haemorrhage where media clarity permits. This may prevent the need for extensive investigations, for example, to exclude underlying vasculitic, septic or haematological disorders [5].

Further studies are required to determine the prevalence of persistent hyaloid remnants with proven flow signals using OCT-A in paediatric populations, and to further validate the possible association with vitreous haemorrhage.
